# Comparison of hypertension healthcare outcomes among older people in the USA and England

**DOI:** 10.1136/jech-2014-205336

**Published:** 2015-11-23

**Authors:** Alan Marshall, James Nazroo, Kevin Feeney, Jinkook Lee, Bram Vanhoutte, Neil Pendleton

**Affiliations:** 1 Department of Geography and Sustainable Development, University of St Andrews, St Andrews, Fife, UK; 2 Cathie Marsh Institute for Social Research, University of Manchester, Manchester, UK; 3 RAND Corporation, Santa Monica, California, USA; 4 Department of Demography, University of California, Berkeley, California, USA; 5 University of Southern California, Davis School of Gerontology, Los Angeles, California, USA; 6 Institute of Brain, Behaviour and Mental Health, Clinical Sciences Building, Salford Royal NHS Foundation Trust, Stott Lane, Salford, UK

**Keywords:** ACCESS TO HLTH CARE, Health inequalities, PUBLIC HEALTH

## Abstract

**Background:**

The USA and England have very different health systems. Comparing hypertension care outcomes in each country enables an evaluation of the effectiveness of each system.

**Method:**

The English Longitudinal Study of Ageing and the Health and Retirement Survey are used to compare the prevalence of controlled, uncontrolled and undiagnosed hypertension within the hypertensive population (diagnosed or measured within the survey data used) aged 50 years and above in the USA and in England.

**Results:**

Controlled hypertension is more prevalent within the hypertensive population in the USA (age 50–64: 0.53 (0.50 to 0.57) and age 65+: 0.51 (0.49 to 0.53)) than in England (age 50–64: 0.45 (0.42 to 0.48) and age 65+: 0.42 (0.40 to 0.45)). This difference is driven by lower undiagnosed hypertension in the USA (age 50–64: 0.18 (0.15–0.21) and age 65+: 0.13 (0.12 to 0.14)) relative to England (age 50–64: 0.26 (0.24 to 0.29) and age 65+: 0.22 (0.20 to 0.24)). The prevalence of uncontrolled hypertension within the hypertensive population is very similar in the USA (age 50–64: 0.29 (0.26 to 0.32) and age 65+: 0.36 (0.34 to 0.38)) and England (age 50–64: 0.29 (0.26 to 0.32) and age 65+: 0.36 (0.34 to 0.39)). Hypertension care outcomes are comparable across US insurance categories. In both countries, undiagnosed hypertension is positively correlated with wealth (ages 50–64). Uncontrolled hypertension declines with rising wealth in the USA.

**Conclusions:**

Different diagnostic practices are likely to drive the cross-country differences in undiagnosed hypertension. US government health systems perform at least as well as private healthcare and are more equitable in the distribution of care outcomes. Higher undiagnosed hypertension among the affluent may reflect less frequent medical contact.

## Introduction

Hypertension is a major risk factor for cardiovascular disease.^1^ Recent increases in the hypertensive population are attributed to population ageing, population growth and the rise of unhealthy lifestyle choices related to diet and sedentary living.^2^ In developed countries, hypertension is most prevalent at the middle and older ages and is predominantly systolic in nature.^3^ Hypertension increases with age because of increases in vascular resistance and arterial stiffness^4^ with aspects of culture and environment being important contributory factors.^5^ Control of hypertension, through healthy lifestyle choices and, where necessary, medical intervention, is recognised as a key way to mitigate the healthcare challenges associated with population ageing.^6–9^

In this paper, we compare hypertension healthcare outcomes among older people (aged 50 years and above) in the USA and England. We focus on the total hypertensive population (either diagnosed with hypertension or measured as hypertensive), assessing whether levels of controlled, uncontrolled and undiagnosed hypertension among older people differ in the USA and England. We also stratify our analysis by wealth and (US) health insurance. Control of hypertension provides interesting insights into the effectiveness of a particular healthcare system. Hypertension is usually asymptomatic and so identification requires effective screening, but the condition is relatively easy to treat and interventions are cheaper than dealing with subsequent heath problems.^1^ Our overall concern is to compare outcomes across different healthcare systems and populations served by them.

The provision of healthcare is very different in the USA and England. In England, the National Health Service provides a publicly funded healthcare system with a comprehensive range of health services, most of which are free at the point of use. In contrast, the USA lacks universal health coverage and most healthcare is provided through private organisations. There are two main Government health insurance schemes. Medicaid provides health insurance coverage to the poor, disabled and those with specific health problems. Medicare covers all those aged over 65 and who are US citizens or have been permanent residents in the USA for at least 5 years.

The guidelines around the diagnosis and treatment of hypertension are more aggressive in the USA than in England.^10^ ^11^ While both countries use a clinical threshold of 140/90 mm Hg, the USA also identifies a prehypertensive group (120–139 mm Hg) who are encouraged to make lifestyle changes to reduce blood pressure.^11^ In England (but not the USA), a formal cardiovascular risk assessment precedes hypertension treatment. In the absence of cardiovascular risks or existing organ damage, treatment is then offered to those with measured blood pressure greater than 160/110 mm Hg. The Quality Outcomes Framework in England uses an auditing blood pressure threshold of 150/90 mm Hg, rather than 140/90 mm Hg as in the National Institute for Care Excellence clinical guidelines. Higher levels of self-reported doctor-diagnosed hypertension in older adults (age 55–64) in the USA (42%) compared to England (34%)^12^ and greater use of medication to control hypertension^13^ ^14^ in the USA reflect the different guidelines described.

The existing research on hypertension care in the USA and England focuses on management of the condition in the population diagnosed with hypertension. It finds no cross-country difference in the levels of control of hypertension to clinical targets between the ages of 50 and 65 but greater control in the USA over the age of 65.^14^ We extend this research in a number of important areas, providing a fuller account of the effectiveness of each healthcare system in managing the condition. We include undiagnosed hypertension in our analysis rather than focusing only on the management of known cases of high blood pressure. Public health systems have a responsibility to treat known cases of hypertension as well as to identify individuals with high blood pressure, especially given the asymptomatic nature of the condition. Thus, comparison of undiagnosed hypertension in the USA and England is an essential aspect of any cross-national evaluation of hypertension management. We also consider differences in hypertension care outcomes among persons aged 50–64 years with different forms of US health insurance, rather than simply treating this age group as being covered by a single ‘market-based’ system.^14^ This distinction is particularly valuable because it includes those individuals who hold no health insurance, an important concern of healthcare systems dominated by the private sector.

## Methods

### Data

We use wave four of the English Longitudinal Study of Ageing (ELSA)^15^ and wave nine of the Health and Retirement Survey (HRS),^16^ representative samples of the population aged over 50 in England and the USA in 2008/2009. We include only those previously diagnosed with or being treated for hypertension or who were measured as hypertensive; all our results (unless otherwise stated) apply to this hypertensive population. Our sample sizes are 4586 in HRS and 4307 in ELSA. Table 1 provides the descriptive statistics.

**Table 1:** Summary statistics on variables in the HRS and ELSA samples

	England			USA		
	N	Per cent	95% CI	N	Per cent	95% CI
Hypertension care outcome						
Hypertension controlled	1849	44	42 to 45	2381	51	49 to 53
Hypertension uncontrolled	1428	33	31 to 34	1563	33	32 to 35
Hypertension undiagnosed	1030	24	22 to 25	642	16	14 to 17
Gender						
Males	2012	47	46 to 49	1887	47	45 to 49
Females	2325	53	51 to 54	2650	53	51 to 55
Age†						
50–54	272	8	7 to 9	148	2	1 to 3
55–59	612	18	16 to 19	548	22	21 to 24
60–64	900	17	16 to 18	558	20	18 to 22
65–69	682	13	12 to 14	875	16	15 to 17
70–74	764	14	13 to 15	874	13	11 to 13
75–79	527	12	11 to 14	705	11	10 to 12
80–84	326	9	8 to 11	441	9	8 to 10
85	254	8	7 to 10	388	7	7 to 8
BMI						
Underweight	21	0.01	0.003 to 0.009	47	0.009	0.006 to 0.012
Normal	817	20	18 to 21	1121	24	22 to 25
Overweight	1716	42	40 to 44	1730	39	37 to 41
Obese	1570	38	36 to 40	1591	37	35 to 39
Ethnicity						
White	4202	97	96 to 98	3592	84	82 to 85
Non White	102	3	2 to 4	945	16	15 to 18
Wealth quintiles						
Least well off	767	21	20 to 23	894	19	18 to 21
Second least well off	886	22	20 to 23	1015	22	21 to 24
Middle wealth quintile	857	19	18 to 21	916	20	18 to 21
Second most well off	890	20	19 to 22	876	19	18 to 21
Most well off	846	17	16 to 19	836	19	18 to 21
Health Insurance						
Private insurance	NA			2558	63	61 to 64
Government insurance	NA			1778	32	30 to 34
No insurance	NA			190	6	5 to 7
Blood pressure test						
Took a test in the last year	3883	88	87 to 89	NA		
Did not take a test in the last year	500	12	11 to 13	NA		

All percentages reported in this table are weighted using appropriate survey weights.

BMI, body mass index; ELSA, English Longitudinal Study of Ageing; HRS, Health and Retirement Survey; NA, not applicable.

### Variables

HRS and ELSA are harmonised with a comparable set of data on the health and circumstances of the older population. We create a hypertension care variable with three outcomes:

- *Hypertension controlled*—individuals diagnosed with hypertension in the past or receiving treatment for the condition who were normotensive in the survey
- *Hypertension uncontrolled*—individuals diagnosed with hypertension in the past or receiving treatment for the condition who were hypertensive in the survey
- *Hypertension undiagnosed*—individuals never diagnosed with hypertension in the past and not receiving treatment for the condition who were hypertensive in the survey

Throughout this paper, we use a threshold of systolic blood pressure over 140 mm Hg or a diastolic blood pressure over 90 mm Hg to indicate measured high blood pressure reflecting clinical guidelines. In the HRS and ELSA, three blood pressure measurements were taken 60 s apart. The average of at least two of these measures is used in the analysis reported here. Blood pressure was measured using an Omron HEM-780 blood pressure monitor in the HRS and an Omron HEM-907 blood pressure monitor in ELSA. In HRS and ELSA, previous diagnosis and hypertension treatment are self-reported. We include a number of other control variables in our model that are known to be associated with hypertension (age, sex, body mass index (BMI), ethnicity (White, Non-White England, Non-White US), wealth quintiles (total net wealth of household (US) or benefit unit (England)) and US insurance group (no insurance, private insurance, government insurance).

### Model

We fit multinomial logistic regression models to predict hypertension healthcare outcome taking hypertension controlled as our reference category. We fit three sets of models.

1. Base model—country, age, sex, BMI, ethnicity (White, England Non-White, US Non-White)
2. Health insurance model—country and insurance status (England, US private insurance, US government insurance, US no insurance), age, sex, ethnicity, BMI, wealth
3. Wealth inequality model—age, sex, BMI, ethnicity and wealth (models fitted for the USA and England separately)

We fit all models in Stata (SE/12.1 for windows) using maximum likelihood estimation and use appropriate survey weights to account for sample selection and non-response. We fit separate models for the population aged under 65 and the population aged 65 or over, reflecting the very different health insurance systems in the USA for each age group.

## Results

**Figure 1:**
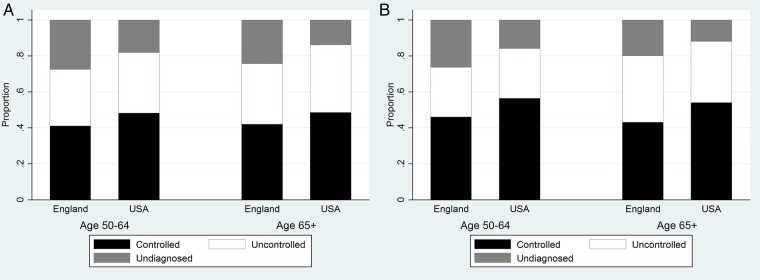
(A) Proportions in each hypertension category (hypertensive controlled, hypertensive uncontrolled and hypertensive undiagnosed) in England and the USA (males) (B). Proportions in each hypertension category (hypertensive controlled, hypertensive uncontrolled and hypertensive undiagnosed) in England and the USA (females).

For the full older population in the HRS and ELSA samples, the mean systolic blood pressure is higher in England (133 mm Hg) compared to the USA (131 mm Hg) (p<0.0001), a difference that holds for women (USA: 132 mm Hg, England 130 mm Hg (p<0.0001)), but not men (USA: 134 mm Hg, England 134 mm Hg (p=0.94)). Mean diastolic blood pressure is lower in England (74 mm Hg) relative to the USA (79 mm Hg) (p<0.0001) with similar findings by sex. The analysis presented from here relates to the population either previously treated/diagnosed with hypertension or measured with high blood pressure. In the USA, this comprises 59% of the population aged 50–64 and 74% of the population aged 65+; in England, this comprises 45% of the population aged 50–64 and 68% of the population aged 65+. These differences between countries for each age group are statistically significant (p<0.0001) and driven by higher levels of diagnosis/treatment in the USA. Figure 1 summarises these differences, with the proportion of the total hypertensive population with uncontrolled hypertension comparable in each country.

We now consider whether the cross-country differences in hypertension care outcomes (figure 1) hold after controlling for age, sex, BMI and ethnicity. We then examine if there are further differences in hypertension care outcomes across US insurance groups and how these compare to England where there is universal access to the National Health Service.

**Figure 2:**
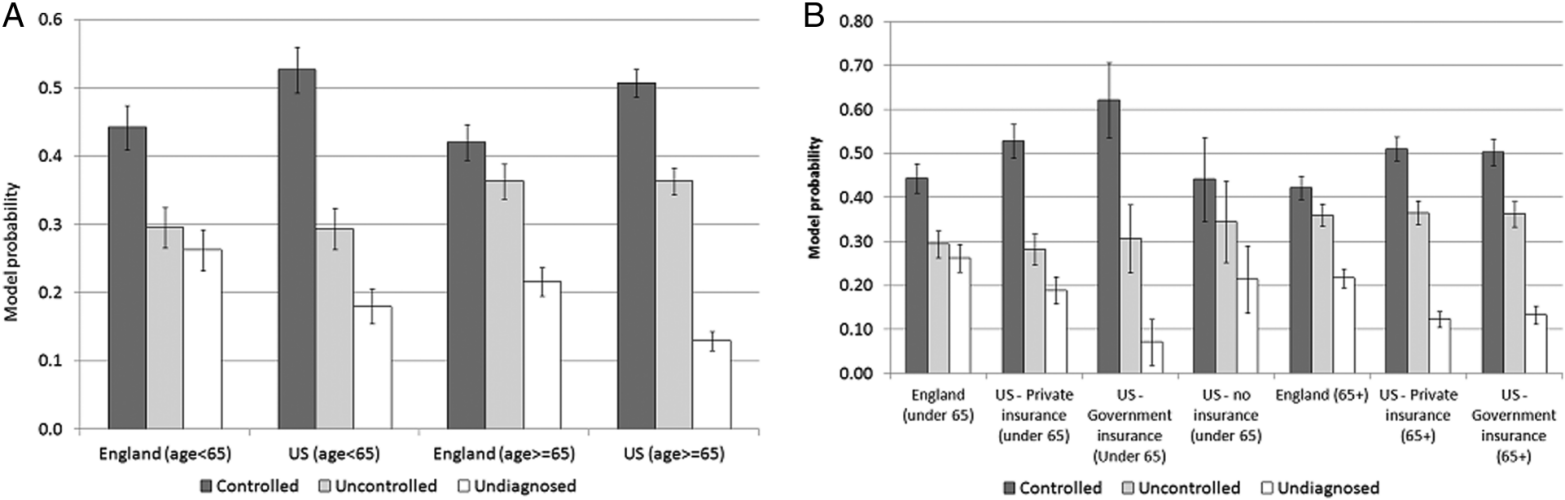
(A) Model probabilities of hypertension care outcomes in the USA and England in the base model (B). Model probabilities of hypertension care outcomes in the USA and England in the insurance model. The health insurance model includes explanatory variables of age, sex, wealth, ethnicity (white, US Non-White, England Non-White), BMI and insurance status (US private insurance, US Government insurance, US no insurance and England (NHS)). BMI, body mass index; NHS, National Health Service*.*

Taking the base model first, the most striking difference in figure 2A is the lower predicted probability of having undiagnosed hypertension in the USA compared to England for both the 50–64 and 65+ age groups. Figure 2A reveals higher control of hypertension in the USA compared to England (at both age groups) whilst levels of uncontrolled hypertension are very similar in the USA and England at each age group. Interestingly, if we set the threshold for measured high blood pressure at the auditing level in the English Quality Outcomes Framework (150/90 mm Hg), then cross-national differences in care outcomes are not significant except for persisting (but attenuated) lower levels of undiagnosed hypertension in the USA at the very oldest ages (results not shown).

The insurance model adds detail to the base model by subdividing the USA into groups of ‘private insurance’, ‘Government insurance’ and ‘no insurance’ (see table 2 and figure 2B.). Striking results are the very low level of undiagnosed hypertension among those with government insurance (50–64) and the comparability of hypertension healthcare outcomes for those over the age of 65 in the USA regardless of the insurance category. Unsurprisingly, those with no insurance have the worst care outcomes in the USA although the relative risk ratios are not significantly different to the private group (reference category) indicating no difference in the relative risk of switching between categories of hypertension care conditional on being in the ‘US no insurance group’ compared to the ‘US private insurance group’. However, it seems likely that the small sample size of the population with no insurance (see table 1) lacks sufficient power for differences to be distinguished. Levels of undiagnosed hypertension are significantly higher in England compared to the US group with private insurance, but model probabilities of uncontrolled hypertension are comparable in England and for those in the US with private insurance.

**Table 2:** RRR* from the base and health insurance models

	Under 65‡			Over 65‡		
	RRR	SE	p>z	RRR	SE	p>z
*Uncontrolled hypertension*†						
Base model§						
England	1			1		
USA	0.83	0.10	0.11	0.83	0.06	0.02
Insurance model¶						
US Private insurance	1			1		
US Government insurance	0.96	0.20	0.86	1.02	0.09	0.80
US no insurance	1.54	0.38	0.07	NA	NA	NA
England	1.25	0.15	0.07	1.21	0.10	0.03
*Undiagnosed hypertension*†						
Base model§						
England	1			1		
USA	0.57	0.07	<0.0001	0.50	0.05	<0.0001
Insurance model¶						
US Private insurance	1			1		
US Government insurance	0.32	0.13	0.01	1.11	0.15	0.45
US no insurance	1.35	0.37	0.27	NA	NA	NA
England	1.66	0.22	<0.0001	2.14	0.24	<0.0001

*RRR are the exponential of the coefficients in the multinomial logistic regression. They give the relative risk of switching between explanatory variable categories on being in a category of the response variable compared to the reference category. For example, in the insurance model (under the age of 65), the relative risk ratio of switching from US private insurance to US Government insurance is 0.32 for being in the undiagnosed hypertension category versus the controlled hypertension category (reference). In other words, the expected risk of staying in the undiagnosed hypertension category (as opposed to the reference of controlled hypertension) is lower for respondents with US Government insurance than with US Private insurance.

†The reference category is controlled hypertension.

‡Models are fitted for the under 65 and the 65 and over age groups separately.

§The base model includes explanatory variables of age, sex, country, ethnicity (white, US Non-White, England Non-White), BMI.

¶The health insurance model includes explanatory variables of age, sex, wealth, ethnicity (white, US Non-White, England Non-White), BMI and insurance status (US private insurance, US Government insurance, US no insurance and England (NHS)).

BMI, body mass index; NA, not applicable; NHS, National Health Service; RRR, relative risk ratios.

The wealth models test whether there are wealth gradients in hypertension healthcare outcomes in the USA and England separately.^i^ There is little evidence of a wealth gradient in uncontrolled hypertension in England, but an indication of lower risks of uncontrolled hypertension with increasing wealth in the USA (see table 3). For the 50–64 age group in England and the USA, there is a suggestion of increases in the probability of undiagnosed hypertension with wealth. Interestingly, the wealth gradient of undiagnosed hypertension in England disappears after including a variable indicating whether an individual had a blood pressure test in the year prior to the interview (results not shown). We do not find any evidence for a wealth gradient in undiagnosed hypertension for the 65+ age group in either country.

**Table 3:** RRR* from the USA and England wealth models

	Under 65			Over 65		
	RRR	SE	p>z	RRR	SE	p>z
*England wealth model*†						
Uncontrolled hypertension‡						
Wealth model (England)						
1 Poorest quintile	1			1		
2	0.87	0.21	0.56	1.27	0.25	0.23
3	1.00	0.25	1.00	1.15	0.22	0.47
4	1.56	0.36	0.05	1.25	0.25	0.26
5 Richest quintile	1.08	0.25	0.76	1.29	0.26	0.20
Undiagnosed hypertension‡						
Wealth model (England)						
1 Poorest quintile	1			1		
2	1.19	0.29	0.47	0.89	0.19	0.58
3	1.46	0.37	0.13	0.92	0.20	0.71
4	1.68	0.41	0.03	0.96	0.21	0.84
5 Richest quintile	1.58	0.38	0.06	1.36	0.29	0.15
*US wealth model*†						
Uncontrolled hypertension‡						
Wealth model (USA)						
1 Poorest quintile	1			1		
2	1.07	0.23	0.75	0.98	0.14	0.91
3	0.80	0.19	0.34	1.00	0.14	0.98
4	0.65	0.17	0.10	0.73	0.11	0.03
5 Richest quintile	0.57	0.15	0.04	0.78	0.12	0.10
Undiagnosed hypertension‡						
Wealth model (USA)						
1 Poorest quintile	1			1		
2	1.58	0.48	0.13	1.07	0.23	0.77
3	1.46	0.47	0.24	1.21	0.26	0.39
4	2.26	0.72	0.01	1.24	0.26	0.30
5 Richest quintile	2.43	0.77	0.01	1.07	0.23	0.76

*The relative risk ratios are the exponential of the coefficients in the multinomial logistic regression. They give the relative risk of switching between explanatory variable categories on being in a category of the response variable compared to the reference category. For example, in the wealth model (under the age of 65), the relative risk ratio of switching from the poorest quintile to the richest quintile is 0.57 for being in the uncontrolled hypertension category versus the controlled hypertension category (reference). In other words, the expected risk of staying in the uncontrolled hypertension category (as opposed to the reference of controlled hypertension) is lower for the richest individuals compared to the poorest individuals.

†Models control for age, sex, wealth, ethnicity (white, Non-White), BMI and are fitted separately for each country and for each age groups (50–64 and 65+).

‡The reference category is controlled hypertension.

BMI, body mass index; RRR, relative risk ratios.

## Discussion

The results above reveal four key findings relating to the population with hypertension (either previously diagnosed or measured within the HRS/ELSA surveys). First, there are higher levels of undiagnosed hypertension in England relative to the USA with comparable probabilities of uncontrolled hypertension in each country. Second, there is no evidence to suggest that older people in the USA with private health insurance receive better hypertension care compared to those who hold government insurance, including at the 50–64 age group, where there is a suggestion that care outcomes are worst for those lacking any health insurance. Third, in each country, there are higher levels of undiagnosed hypertension among the most affluent than among the poorest. Finally, the US healthcare system is less equitable than that in England in terms of uncontrolled hypertension, which becomes less prevalent with increasing wealth.

The greater extent of undiagnosed hypertension (among those with hypertension) in England relative to the USA may stem from the different procedures in each healthcare system for diagnosing and treating hypertension. In particular, the Quality Outcomes Framework in England may have incentivised primary care practices to treat to a blood pressure threshold of 150/90 mm Hg, rather than 140/90 mm Hg as in the National Institute for Care Excellence clinical guidelines. Evidence on the benefits of medication for those with borderline hypertension (140–150 mm Hg) is mixed,^17^ particularly at the very oldest ages (80+),^7^ ^18^ and further research is required to determine whether the lower levels of undiagnosed hypertension in the USA confer cardiovascular advantages.

In the USA, the private healthcare system is not more effective than government healthcare systems in managing hypertension among the elderly. The only significant difference between care outcomes is the lower level of undiagnosed hypertension for those between 50 and 64 years of age with government insurance. Those holding government insurance at these ages are typically either living in poverty or have poor health qualifying them for Medicaid. We argue that those with Medicaid are likely to have greater contact with health professionals as a result of their health status, conferring increased opportunity for hypertension diagnosis. Those with no health insurance, unsurprisingly, have the worst hypertension healthcare outcomes in the USA. Although the poorer care outcome for those with no insurance failed to achieve statistical significance, it seems likely that the small sample size (192) and a lack of power is most likely responsible for this lack of significance.

The increasing risk of undiagnosed hypertension with wealth in the USA and England is an interesting finding. We argue that a key driver of this gradient in undiagnosed hypertension is the frequency with which poorer individuals see health professionals. Research suggests poorer people are most likely to visit their doctor (although this gradient loses statistical significant or changes direction when health need is taken into account),^19^ offering a greater opportunity for blood pressure checks. Support for our hypothesis is provided by including a variable indicating whether a person had their blood pressure measured in the year prior to their survey interview. This removes the wealth gradient in undiagnosed hypertension in England (results not shown). Undiagnosed hypertension is not related to wealth at the older age group (65+), possibly reflecting the introduction of routine health tests at age 65.

Levels of uncontrolled hypertension are more evenly distributed by wealth in universal government administered healthcare systems than in private healthcare systems. We see no evidence of a gradient in uncontrolled hypertension in England under the NHS. In the USA, the risks of uncontrolled hypertension fall with wealth for those aged 50–64, where private health insurance dominates, and while a similar gradient exists over the age of 65, where there is universal health coverage (Medicare), it is less prominent. Private healthcare systems appear to disadvantage poorer people who may be unable to afford the costs of healthcare and have higher levels of uncontrolled hypertension relative to the richest as a result, a finding noted elsewhere.^14^

A key strength of this study is that our findings are based on representative samples of the community-dwelling population at least 50 years of age in the USA and England. However, there are some limitations to this work. First, our data are subject to issues of non-response. Although we deal with any resulting bias through the use of survey weights and refreshment samples, we cannot be certain that we have accounted for all the differences between those who participated in the survey and those who dropped out. Second, we attribute differences in healthcare outcomes to the provider of healthcare in each country (government and private) but acknowledge that a number of other differences might be involved. For example, compared to England, the USA has a more specialised health service (higher ratio of specialists to generalists), spends more of its gross domestic product (GDP) on healthcare, and has a multitude of investors in healthcare (compared to a single payer in England), complicating chains of accountability.^14^ Finally, we accept that our measurement threshold for hypertension of 140/90 mm Hg on average of at least two measurements during a single clinical visit differs slightly from the national guidelines in the USA and England,^10^ ^11^ which require demonstration of high blood pressure on two separate clinical occasions. As a result, our analysis may overestimate levels of undiagnosed hypertension. Nevertheless, given that we used common study methods and definitions in the USA and England, our findings on country-specific differences in hypertension care outcomes should still hold.

In conclusion, our results on hypertension care outcomes for the hypertensive population (diagnosed or measured within the HRS/ELSA survey) reveal higher levels of undiagnosed hypertension in England compared to the USA, a result that may stem from different guidelines around the treatment of the condition. Importantly, levels of uncontrolled hypertension are similar in each country, indicating that once the condition is identified, the performance of the USA and English health systems is comparable. The US health system appears less equitable than that in England in terms of uncontrolled hypertension, perhaps as a result of the costs of healthcare in the USA which poorer individuals are less able to meet. For older people in the USA, there appears to be no advantage in holding private health insurance compared to Government health insurance in terms of the hypertension care received. Interestingly, in both countries, the levels of undiagnosed hypertension are greatest for the most affluent, a result that may reflect less frequent tests of blood pressure among the most wealthy.

**What is already known on this subject**

- Previous research suggests that treatment of hypertension among the adult population is more aggressive in the USA compared to the UK. At the older ages, levels of diagnosed hypertension are higher in the USA compared to England with comparable levels of measured hypertension in each country (using a threshold of 140/90 mm Hg).

**What this study adds**

- We extend the existing research to examine differences in hypertension healthcare outcomes across wealth quintiles and insurance groups among older populations in the USA and in England. In our analysis of a sample of the population with hypertension (either diagnosed or measured within the survey data used), we confirm the consequences of more aggressive treatment of hypertension in the USA, finding higher levels of undiagnosed hypertension among older people in England that may stem from different guidelines around the treatment of the condition in each country. We do not find evidence of better hypertension healthcare in government compared to private health insurance groups in the USA. In each country, there is some evidence that levels of undiagnosed hypertension rise with wealth for those under 65, most likely reflecting wealth gradients in frequency of blood pressure measurement. In terms of uncontrolled hypertension, we find evidence to suggest that Government health systems lead to more equitable care compared to private health systems. Uncontrolled hypertension reduces with increasing wealth in the USA, particularly for the 50–64 age group where the majority of healthcare is provided by the private sector. We observe no such gradient in uncontrolled hypertension in England where the National Health Service provides universal health cover.

## Footnotes

i We obtain the same conclusions if we fit a single model with a country*wealth interaction (results not shown).
